# Datasets of productivity and vegetation composition of boreal stands from an experiment comparing silviculture scenarios of increasing intensity after 20 years

**DOI:** 10.1016/j.dib.2022.108387

**Published:** 2022-06-14

**Authors:** Morgane Urli, Nelson Thiffault, Daniel Chalifour

**Affiliations:** a Centre de Recherche et d'Innovation Sur Les Végétaux, Département des Sols et de Génie Agroalimentaire, Université Laval, 2480 boul. Hochelaga, Quebec, QC G1V 0A6, Canada; b Canadian Wood Fibre Centre, Canadian Forest Service, Natural Resources Canada, 1055, du P.E.P.S., P.O. Box 10380, Sainte-Foy Stn., Quebec, QC G1V 4C7, Canada; c Centre for Forest Research, Faculty of Forestry, Geography and Geomatics, Université Laval, 2405 rue de la Terrasse, Quebec, QC G1V 0A6, Canada; d Société de Protection des Forêts Contre le Feu, Base de Maniwaki, 176 route 105, Messines, QC J0X 2J0, Canada

**Keywords:** Vegetation diversity, Ecosystem-based management, Silviculture, Forest composition

## Abstract

This data article describes datasets of plant community composition, dendrometric measurements, quantity and quality of snags of humid boreal stands (Quebec, Canada) from an experiment comparing silviculture scenarios of increasing intensity: (i) careful logging around advance growth (CLAAG); (ii) CLAAG followed by pre-commercial thinning; (iii) plantation followed by mechanical release; and (iv) plantation followed by chemical release and within five naturally disturbed sites. These data enable researchers to examine vegetation biodiversity recovery, ecosystem variables such as dead wood, and boreal stand productivity 20 years following the start of increasing-intensity silviculture scenarios. As a result, these data can be used to investigate the trade-off between keeping important ecosystem aspects of natural forests and maintaining and/or growing merchantable wood production at the stand level. This trade-off is the paradigm of forest ecosystem-based management, which aims to reduce the ecological distance between natural and managed forests in order to balance ecological challenges with the provision of socioeconomic services.

## Specifications Table

**Table:** 

Subject	Biological Sciences
Specific subject area	Forestry and Plant Science
Type of data	Tables and R code
How the data were acquired	In July 2009, 20 years after the onset of the silviculture scenarios, we conducted vegetation surveys (composition and dendrometric measurements) of four circular 100 m^2^ plots within five naturally disturbed sites and 20 managed sites representing a gradient of increasing silviculture intensity, (i) careful logging around advance growth (CLAAG); (ii) CLAAG followed by pre-commercial thinning; (iii) plantation followed by mechanical release of crop trees using motor-manual brushsaws; and (iv) plantation followed by chemical release of crop trees using herbicide.
Data format	Clean data and analysed data
Description of data collection	Vegetation surveys were conducted in July 2009 in humid boreal stands from an experimental design comparing silviculture scenarios of increasing intensity: (i) careful logging around advance growth (CLAAG); (ii) CLAAG followed by pre-commercial thinning; (iii) plantation followed by mechanical release of crop trees using motor-manual brushsaws; and (iv) plantation followed by chemical release of crop trees using herbicide and within five naturally disturbed sites. The surveys comprise data about the density of snags and of living trees (defined as having a diameter at breast height (1.3 m), DBH ≥ 1.1 cm), dendrometric measurements (DBH, height and basal area) of conifer and deciduous species, percent cover of trees and high regeneration (defined as having a height ≥ 60 cm and a DBH < 1 cm), shrub (defined as having a height < 60 cm), herbaceous species and various taxonomic groups (ferns, mosses, sphagnum, lichens, grasses and *Lycopodium L.*). Data on the ratio of intercepted light to full sunlight conditions averaged by plot, derived from Photosynthetic photon flux density (PPFD) readings, are also provided.
Data source location	Forêt Montmorency and Parc de la Jacques-Cartier, both located about 80 km North of Quebec City, Canada (47°16’–47°21’N; 71°01’–71°19’W, cf. Fig. 1 of Urli et al. 2017).
Data accessibility	Repository name: Figshare Data identification number: https://doi.org/10.6084/m9.figshare.18785699.v1 Direct URL to data: https://figshare.com/articles/dataset/Datasets_of_productivity_and_vegetation_composition_of_boreal_stands_from_an_experimental_design_comparing_silviculture_scenarios_of_increasing_intensity_after_20_years/18785699/3
Related research article	Urli et al. Key ecosystem attributes and productivity of boreal stands 20 years after the onset of silviculture scenarios of increasing intensity, For. Ecol. Manage. 389 (2017) 404–416. https://doi.org/10.1016/j.foreco.2017.01.007

**Value of the Data**

• These data allow the study of vegetation biodiversity recovery and ecosystem attributes such as dead wood and productivity of boreal stands, 20 years after the onset of silviculture scenarios of increasing intensity. These data are useful to study the trade-off between preserving critical ecosystem features of natural forests and maintaining and/or increasing merchantable wood production at the stand level. This trade-off is the paradigm of forest ecosystem-based management aiming at minimising the ecological distance between natural and managed forests in order to reconcile ecological challenges with the provision of socioeconomic services.
• Researchers and forest practitioners may benefit from these data.
• These data can be used in meta-analyses, along with other similar vegetation surveys, to study post-harvest vegetation biodiversity and stand attributes of boreal forests in the long term and to compare various silviculture scenarios.

## 1 Data Description

These data, from clean to analysed, cover different vegetation surveys (Table 1). All metadata, as well as the name of species and description of each variables, are provided in the Metadata.txt file.

**Table 1:** Description of each data file.

Name of the file	Description of the file
DBH_cleandata.csv	DBH per individual tree (cm)
Dominant_tree_cleandata.csv	DBH (cm), ground level diameter (cm) and height (m) of two conifers and two deciduous trees (when possible) representative of the dominant or codominant crown classes within the upper canopy
Snag_cleandata.csv	DBH per individual snag (cm)
PPFD_cleandata.csv	PPFD (micromol. m^−2^ s^−1^) at 1 m height, PPFD (micromol. m^−2^ s^−1^) in an open area located near the plots and ratio of PPFD at 1 m height to PPFD in an open area of the 4 cardinal points per plot
Tree.csv	Density of stems of living trees per plot (stems 100 m^2^), calculated from data of DBH_cleandata.csv
High_Regeneration.csv	Density of high regeneration per plot (stems 100 m^2^)
Shrub.csv	Percent cover of shrub per plot (10% classes)
Herbaceous.csv	Percent cover of herbaceous species per plot (10% classes)
Taxonomical_groups.csv	Percent cover of taxonomical groups (Bryophyta, Sphagnum, Lichen, Poaceae, Filocophyta, Latifolie, Lycopodiales) per plot (10% classes)
Tree_cover.csv	Percent total cover of trees per plot (10% classes)
DBH_plot.csv	Mean, standard deviation, standard error of DBH (1.3 m) of conifer and deciduous species per plot and number of conifer and deciduous species per plot, calculated from data of DBH_cleandata.csv
Cum_BA_plot.csv	Cumulative basal area (m^2^ ha^−1^) of conifer and deciduous species per plot, calculated from data of DBH_cleandata.csv
Snag_plot.csv	Density of conifer and deciduous snags per plot (stems 100 m^−2^), calculated from data of Snag_cleandata.csv
PPFD_plot.csv	Mean, standard deviation, standard error of ratio of Photosynthetic photon flux density (PPFD) at 1 m height to PPFD in an open area, per plot, calculated from data of PPFD_cleandata.csv

## 2 Experimental Design, Materials and Methods

The data were obtained from a replicated field study conducted at Forêt Montmorency and Parc de la Jacques-Cartier, two locations located about about 80 km north of Québec City (Québec, Canada) (cf Fig. 1 from [1]) within the balsam fir (*Abies balsamea* (L.) Mill.)–white birch (*Betula papyrifera* Marsh.) bioclimatic domain described by [2]. Balsam fir, black spruce (*Picea mariana* (Mill.) BSP) and white birch dominate mature forests on mesic sites in this region. The climate is classified as boreal per humid; annual temperature averages 0.5 °C and annual precipitation averages 1583 mm of precipitation, almost two-thirds of which falls as snow (weather station n° 7042388 located at 47°19’N; 71°09’W, [3]). A hilly environment with elevations ranging from 600 to 1100 m, as well as acidic glacial tills, characterize the region. In our study area, spruce budworm (*Choristoneura fumiferana* (Clem.)) outbreaks are the most common natural disturbances, while wildfires are rare [4].

The landscape within Parc de la Jacques-Cartier experienced its last clear-cut harvests in the 1940s and has only been shaped by natural disturbances since. We chose stands within the Parc that experienced a single spruce budworm outbreak between 1974 and 1986. The Forêt Montmorency area is affected by both natural and anthropogenic disturbances. As a result, the forest landscape is characterized by recent clearcuts of up to 25 ha, interspersed by areas of remnant forest (3–10 ha), riparian buffers up to 20 m wide, and roads. Because Forêt Montmorency was sprayed with insecticide during the last outbreak, it was less affected by the spruce budworm infestation than the Parc de la Jacques-Cartier. In Forêt Montmorency, we selected stands that were clear-cut harvested between 1941 and 1944, that were left to naturally regenerate, and that were harvested through careful logging around advanced growth between 1987 and 1989.

We used the experimental setup of [5] to design our experiment. We therefore chose 25 sites, each ranging in size from 6 to 9 ha. Twenty stands were selected within Forêt Montmorency (managed) and five stands were selected within the Parc de la Jacques-Cartier (naturally disturbed). All sites were located on mesic soils, had no remaining mature tree patches, and most supported pure balsam fir stands before disturbance based on forest maps from 1982 to 1983. White birch with balsam fir and black spruce, or only black spruce, dominated the other sites. In 1981, more than two thirds of the sites were 41 to 60 years old and had a relative density of 60–80%. Stands that were affected by the spruce budworm outbreak had densities varying between 40 and 60% and were aged more than 60 years. We examined current and common silviculture scenarios normally applied in these ecosystems. The twenty managed sites resulted from harvesting with careful logging around advance growth (CLAAG) between 1987 and 1989 (cf Figs. 1 and 2 of [1]); they represented four silviculture scenarios of increasing intensity (5 sites each):

(i) No additional silviculture treatments were applied (CLAAG only).
(ii) After CLAAG, we applied pre-commercial thinning in 2000 to reduce conifer density to 1500–3125 stem ha^−1^ (CLAAG + PCT).
(iii) After CLAAG, we scarified the sites in autumn 1989, planted black spruce seedlings in spring 1990 (2000 stems ha^−1^), and mechanically released planted seedlings from competing vegetation over a 1 m radius in 1992 using motor-manual brushsaws (PLM).
(iv) After CLAAG, we applied the same treatments as (iii), but replaced mechanical release by the aerial application of Vision Silviculture herbicide (glyphosate) at a rate of 5 L ha^−1^ (PLC).

In 2000, PLM and PLC sites were subjected to a mechanical juvenile cleaning [6].

In July 2009, vegetation was surveyed on each naturally and disturbed sites. On each site, we established four circular 100 m^2^ plots, with one plot in the centre and the other three plots spaced by 150 m. We established concentric 25 m^2^ subplots in the centre of each 100 m^2^ plot. We measured the density (number of stems per plot) of trees (defined as having a diameter at breast height (1.3 m), DBH ≥ 1.1 cm) and of high regeneration (described as having a height ≥ 60 cm and a DBH < 1 cm) in the 100 m^2^ plots and concentric 25 m^2^ subplots, respectively. DBH was measured for each tree. Using ten percent cover classes, the percent cover of shrubs (height < 60 cm), herbaceous species, and other taxonomic groups (lichens, sphagnum, mosses, grasses, ferns, and *Lycopodium L.*) was visually estimated in each 100 m^2^ plot. In each plot, we measured the height of two conifers and two deciduous trees (when present); measured trees were selected to be representative of the dominant or codominant crown classes. In the 100 m^2^ plots, we also surveyed snags and measured their DBH. Finally, we used a Sunfleck portable ceptometer (Decagon, Devices, Pullman, WA) to quantify the Photosynthetic photon flux density (PPFD, µmol m^-^^2^ s^−1^) at 1 m height at the four cardinal points of each 100 m^2^ plot. We used simultaneous PPFD measurements taken in full sunshine conditions to express the averaged plot-PPFD as a ratio of full available light.

## Ethics Statements

None

## CRediT authorship contribution statement

**Morgane Urli:** Data curation, Formal analysis, Writing – original draft, Writing – review & editing. **Nelson Thiffault:** Conceptualization, Resources, Funding acquisition, Project administration, Writing – review & editing. **Daniel Chalifour:** Funding acquisition, Writing – review & editing.

## Declaration of Competing Interest

The authors declare that they have no known competing financial interests or personal relationships that could have appeared to influence the work reported in this paper.

The authors declare the following financial interests/personal relationships which may be considered as potential competing interests:

## Data Availability

Datasets of productivity and vegetation composition of boreal stands from an experimental design comparing silviculture scenarios of increasing intensity after 20 years (Original data) (Figshare).
